# Implementing an exercise intervention for sarcopenia in oldest-old nursing home residents: protocol for a pragmatic cluster-randomized controlled trial (SCOPE Study)

**DOI:** 10.1186/s12877-026-08002-8

**Published:** 2026-08-31

**Authors:** Cheng Xiang, Lu Li, Jianzhi Zhao, Yuwen Zhou, Wenye Cai, Yixi Guo, Ziru Yang, Xin An, Changxu Gu, Run Ding, Xucheng Wu, Yincun Wang, Di Wang, Shen Xu, Zuyun Liu, Ying Gao

**Affiliations:** 1 Department of Sports Science, College of Education, Zhejiang University, 866 Yuhangtang Rd, Hangzhou, Zhejiang 310058 China; 2 Department of Big Data in Health Science School of Public Health, Center for Clinical Big Data and Analytics Second Affiliated Hospital, Zhejiang Key Laboratory of Intelligent Preventive Medicine, Zhejiang University School of Medicine, 866 Yuhangtang Rd, Hangzhou, Zhejiang 310058 China; 3 School of Physical Education, Hangzhou Normal University, 2318 Yuhangtang Rd, Hangzhou, Zhejiang 311121 China; 4 Department of Geriatrics, Hangzhou TCM Hospital Affiliated to Zhejiang Chinese Medical University, 453 Stadium Road, Hangzhou, Zhejiang 310007 China; 5 Center for Aging & Health Studies, Zhejiang University, Hangzhou, Zhejiang 310058 China

**Keywords:** Sarcopenia, Oldest-old, Baduanjin, Resistance training, Nursing home, Pragmatic randomized controlled trial

## Abstract

**Background:**

Sarcopenia is a progressive age-related condition characterized by loss of muscle mass, strength, and function, with particularly high prevalence among nursing home residents and the oldest-old population. Although exercise is a recognized non-pharmacological intervention, existing evidence has largely been generated in selected community-dwelling populations, limiting its applicability to vulnerable older adults in real-world long-term care settings. The SCOPE study (Sarcopenia Care in Oldest-old: a Pragmatic Exercise cluster-randomized controlled trial) evaluates the effectiveness and feasibility of caregiver-led modified Baduanjin, resistance training, and combined exercise interventions for sarcopenia management in nursing home residents.

**Methods:**

This pragmatic, cluster-randomized controlled trial aims to recruit 104 residents aged ≥ 75 years with sarcopenia from a large nursing home in Hangzhou, China. Participants will be randomized by residential building into four groups: Modified Baduanjin Group, Resistance Training Group, Combined Exercise Group, and Control Group. The 48-week intervention will be delivered by trained caregivers using video-guided sessions, with phased reduction in specialist supervision. The primary outcome is a composite sarcopenia Z-score integrating handgrip strength, Short Physical Performance Battery, and appendicular skeletal muscle mass index. Secondary outcomes include functional mobility, balance, physical activity and sedentary behaviour, neuromuscular activation, questionnaire-based assessments, and saliva biological markers. Clinical events and care-dependency records will also be collected throughout the intervention and follow-up periods. Assessments will be conducted at baseline, week 12, week 24, week 48, and at 1 and 2 years post-intervention.

**Discussion:**

The SCOPE study addresses the need for feasible and sustainable exercise interventions for sarcopenia in real-world nursing home settings. By integrating pragmatic caregiver-led delivery, multimodal outcome assessment, and long-term follow-up, this trial aims to inform exercise prescription, implementation feasibility, and potential mechanisms of adaptation in oldest-old nursing home residents. The findings are expected to support the development of scalable exercise intervention frameworks for sarcopenia management in long-term care facilities.

**Trial registration:**

Chinese Clinical Trial Registry (ChiCTR2500107315).

**Supplementary Information:**

The online version contains supplementary material available at https://doi.org/10.1186/s12877-026-08002-8.

## Background

Sarcopenia is a progressive, age-associated condition characterized by a decline in skeletal muscle mass, strength, and function, a combination that substantially increases the risks of frailty, falls, disability, and mortality [1]. This progressive loss of muscle tissue is estimated to result in up to a 40% reduction in muscle mass between the ages of 20 and 70, with the rate of decline accelerating to approximately 1.4%–2.5% per year after the age of 60 [2]. This age-related decline extends beyond muscle quantity, as muscle strength and physical performance have likewise been shown to deteriorate in a largely linear manner with advancing age [3]. At the population level, this condition affects approximately 10%–16% of older adults worldwide [4], indicating a substantial public health burden. This burden, however, is not evenly distributed across care settings. Evidence from a meta-analysis of 34,955 participants demonstrates that prevalence rates are markedly higher among nursing home residents, reaching 51% (95% CI: 37–66%) in men and 31% (95% CI: 22–42%) in women, whereas substantially lower estimates have been reported among community-dwelling individuals, at 11% (95% CI: 8–13%) in men and 9% (95% CI: 7–11%) in women [5].

With the continued extension of global life expectancy [6], the burden of sarcopenia has increasingly shifted toward adults aged 80 years and above, a population that experiences more pronounced functional decline and a substantially elevated risk of sarcopenia-related adverse events. This concentration of disease burden in the oldest-old, however, is not adequately reflected in the existing evidence base, as most randomized controlled trials (RCT) evaluating behavioral interventions for sarcopenia have been conducted predominantly in relatively younger older adults, typically between 55 and 75 years of age [7, 8]. This mismatch between disease burden and study populations may consequently limit the applicability and clinical relevance of current findings for the oldest-old.

Among modifiable risk factors, physical inactivity has been identified as a key contributor to the development and progression of sarcopenia, primarily through its association with muscle atrophy and functional deterioration [9, 10]. Given this central role, increasing physical activity (PA) has emerged as one of the most effective non-pharmacological strategies to counteract sarcopenia. This therapeutic value is supported by a growing body of evidence, which demonstrates that structured exercise interventions can significantly improve muscle strength, muscle mass, and functional performance in older adults [7, 11–13]. On the basis of this evidence, current clinical guidelines place PA at the core of both sarcopenia prevention and management, particularly in light of the limited availability of effective pharmacological treatments [14, 15].

Resistance training is widely regarded as the cornerstone of exercise-based interventions for sarcopenia [16]. In practice, however, its application among frail or institutionalised older adults is frequently constrained by safety concerns, joint stress, and space limitations [17], underscoring the need for alternative or complementary exercise modalities. In this context, mind–body exercises such as Tai Chi and Baduanjin, which are traditional Chinese practices characterised by slow, flowing movements integrated with breathing and mental focus, have attracted growing interest. Evidence suggests that these low-impact exercises confer benefits across multiple domains, including mental well-being [18], cognitive function [19], balance [20] and physical performance [21] in older adults. Their safety and acceptability, even among frail individuals [22], further highlight their potential value, particularly in promoting neuromuscular coordination [23]. Among these modalities, Baduanjin may be particularly relevant for sarcopenia management because it consists of a limited number of standardised movements, requires no special equipment, imposes relatively low mechanical stress on the joints, and integrates breathing regulation and attentional focus with coordinated trunk and limb movements, postural control, and neuromuscular coordination [24]. Recent RCTs have provided preliminary direct evidence supporting its potential value in sarcopenia management, reporting improvements in physical performance, balance, lower-limb strength, skeletal muscle mass, and other related outcomes in older adults with sarcopenia [25, 26]. Nevertheless, original intervention studies conducted in nursing home settings or specifically targeting the oldest-old remain scarce. Moreover, the standard standing forms of these exercises may remain challenging for the oldest-old, especially for those with marked mobility limitations or a pronounced fear of falling [27].

Although substantial clinical evidence supports the effectiveness of exercise in the management of sarcopenia, much of this evidence has been generated under highly controlled experimental conditions, with limited consideration of implementation in real-world settings such as nursing homes [28]. This gap between efficacy and applicability has prompted calls for a shift in research priorities, emphasising the optimisation of exercise prescriptions and the promotion of long-term adherence [29]. In institutional care environments, intervention success depends not only on clinical efficacy but also on operational feasibility, safety, and the capacity to sustain participant engagement. Accordingly, effective programmes are those that can capitalise on existing facilities and staff resources, minimise dependence on specialised equipment or external expertise, and integrate smoothly into routine care workflows [30].

Against this background, the present pragmatic trial evaluates a caregiver-led exercise programme that integrates modified Baduanjin with resistance training in oldest-old nursing home residents with sarcopenia. Using a cluster-randomised design and multimodal assessment, the study examines the comparative effectiveness of these interventions while simultaneously exploring underlying biological mechanisms and implementation feasibility. By prioritising practicality and sustainability, this programme is intended to provide an exercise framework that can be readily adopted by other long-term care facilities facing similar resource constraints and resident needs.

## Methods

### Sample size determination

The sample size was estimated using G*Power version 3.1 (Heinrich Heine University Düsseldorf, Germany) based on the composite sarcopenia Z-score. A previous comparable trial reported a large intervention effect on sarcopenia Z-score (SMD = 1.40) [31]. With 80% power, a Bonferroni-adjusted two-sided α of 0.0167, and ANCOVA adjustment for baseline sarcopenia Z-score, 16 completers per group are required in an individually randomized design, corresponding to 64 completers in total. To account for cluster randomization at the residential-building level, the sample size was inflated using the adjusted design effect formula, DE = 1 + [m (1 + CV^2^) − 1] × ICC. Based on a recent cluster-randomized trial in senior care facilities using Short Physical Performance Battery (SPPB) as the primary outcome, an intracluster correlation coefficient (ICC) of 0.025 was adopted [32]. A CV of 0.30 was used to account for anticipated variation in cluster size [33]. With 19 clusters, these assumptions yielded an adjusted design effect of approximately 1.12. After allowing for a 30% dropout rate, the sample size was increased to 104 participants, targeting approximately 26 participants per group. Cluster effects will be further addressed using mixed-effects models with random intercepts for residential building and participant.

### Study design and setting

The SCOPE study (Sarcopenia Care in Oldest-old: a Pragmatic Exercise cluster-randomized controlled trial) is a pragmatic cluster-randomized controlled trial, aimed at evaluating the real-world effectiveness and feasibility of different exercise interventions for sarcopenia in nursing home residents aged 75 and above. A total of 104 participants will be randomized into one of four groups: (1) Control Group (CON), (2) Modified Baduanjin Group (MBG), (3) Resistance Training Group (RTG), and (4) Combined Exercise Group (CEG).

**Fig. 1:**
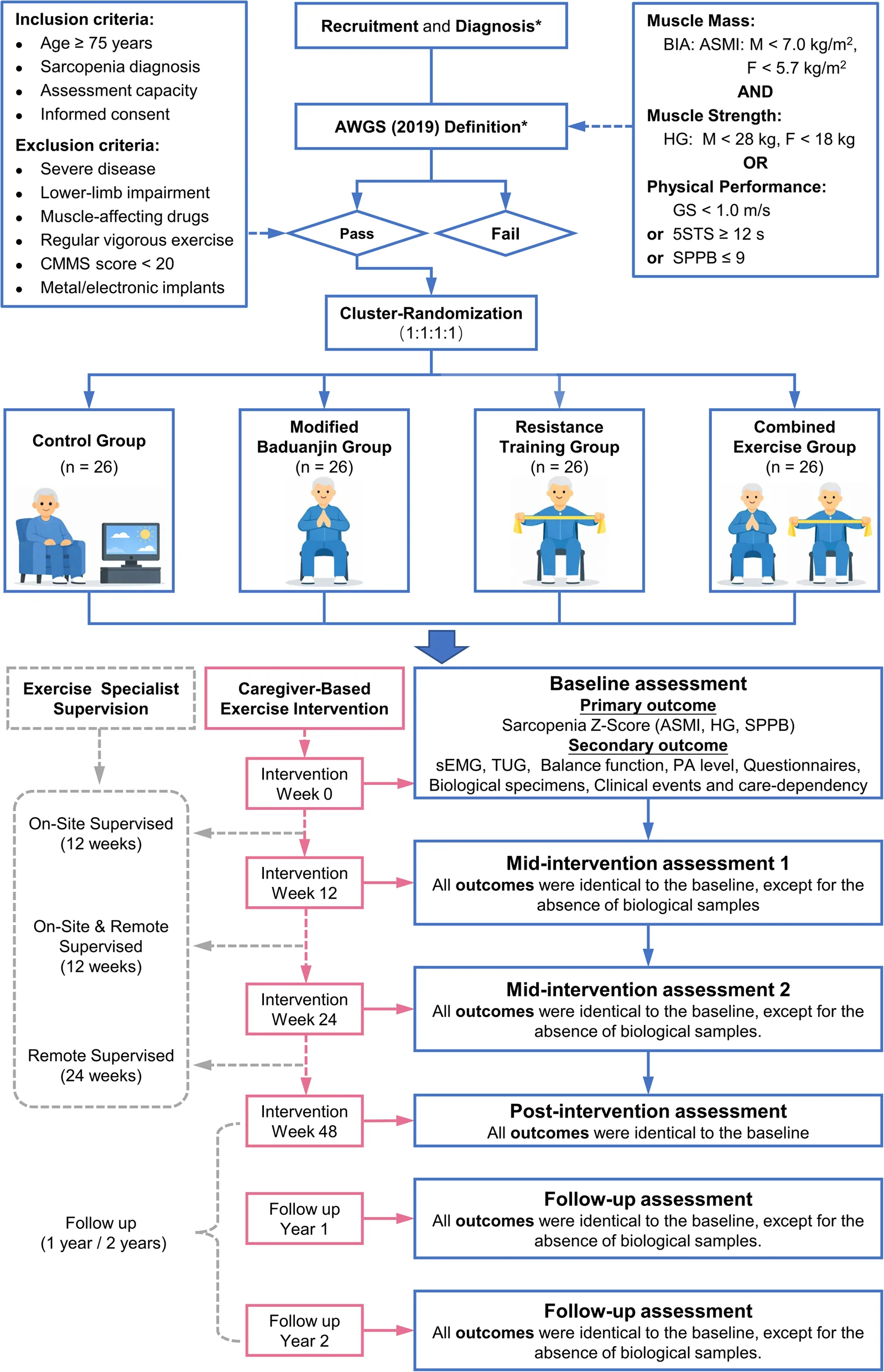
Flow diagram of the SCOPE study. AWGS, Asian Working Group for Sarcopenia; BIA, Bioelectrical Impedance Analysis; ASMI, Appendicular Skeletal Muscle Mass Index; HG, Handgrip Strength; GS, Gait Speed; 5STS, Five-Time Sit-to-Stand Test; SPPB, Short Physical Performance Battery; CMMS, Chinese Mini-Mental Status; TUG, Timed Up and Go Test; PA, Physical Activity

A flow diagram of the trial is presented in Fig. 1. The SCOPE study will begin with continuous screening for sarcopenia following the Asian Working Group for Sarcopenia (AWGS) 2019 criteria [34]. Older adults clinically diagnosed with sarcopenia and meeting the eligibility criteria will be enrolled. After a standardized 30-min health education session and subsequent randomization, participants will undergo a 48-week pragmatic group-based exercise intervention within their residential buildings. This pragmatic intervention will be delivered by trained, familiar caregivers using video materials recorded by exercise specialists, with a phased reduction in researcher supervision over time: full on-site supervision during week 1–12, a hybrid model combining on-site and remote supervision during week 13–24, and fully remote supervision during week 25–48. Outcome assessments will be conducted at baseline, week 12, week 24 and week 48, with additional follow-up evaluations at 1 year and 2 years post intervention to examine the maintenance of long-term effects.

#### Diagnostic criteria of sarcopenia

Sarcopenia is diagnosed according to the AWGS 2019 criteria (the SCOPE study was designed before the AWGS 2025 definition of sarcopenia was published [35]), which require the presence of low muscle mass in combination with low muscle strength and/or low physical performance [34]:

1. Low muscle mass is defined as an appendicular skeletal muscle mass index (ASMI): < 7.0 kg/m^2^ for males and < 5.7 kg/m^2^ for females, measured via bioelectrical impedance analysis (BIA);
2. Low muscle strength is indicated by a handgrip strength (HG) < 28 kg for males and < 18 kg for females;
3. Low physical performance is characterized by a gait speed (GS) < 1.0 m/s, a five-time sit-to-stand test (5STS) ≥ 12 s, or SPPB score ≤ 9.

#### Inclusion criteria

1. Age ≥ 75 years;
2. Diagnosed with sarcopenia according to the criteria described above;
3. Able to understand test instructions, complete questionnaires, and perform the required assessments and exercise movements with or without appropriate assistance;
4. Willing to participate in the study, provide written informed consent, adhere to the intervention protocol, and attend follow-up assessments.

#### Exclusion criteria

1. Severe cardiovascular or cerebrovascular disease, advanced cancer, severe systemic illness, or moderate-to-severe dysfunction of major organs, including the heart, liver, kidneys, or lungs;
2. Lower-limb joint disease, neurological disorder, or functional impairment that prevents safe participation in exercise or required physical assessments;
3. Use within the past 3 months of medications known to substantially affect muscle, bone, or neuromuscular function, such as glucocorticoids, androgens, estrogens, thyroid hormones, or other relevant agents;
4. Regular high-intensity physical exercise before enrollment, defined as ≥ 15 min of vigorous physical activity daily or resistance training at least twice per week for 3 consecutive months;
5. Diagnosed mental disorder or moderate-to-severe cognitive impairment, defined as a Chinese Mini-Mental Status (CMMS) score < 20 (consistent with CMMS severity mapping and cognitive eligibility thresholds used in previous nursing-home exercise trials [36, 37]);
6. Presence of metal or electronic implants that may interfere with body composition assessment or other study examinations.

#### Participant recruitment and screening

Participant recruitment will be conducted in collaboration with the Hangzhou No.3 Social Welfare Institution, the largest nursing home in the region. The institution spans 169 acres and comprises 26 residential buildings with four to six floors each, providing a total capacity of approximately 2,000 beds. The facility serves a population with a mean age exceeding 80 years and is representative of institutionalised older adults in urban China.

To ensure comprehensive outreach, a multi-faceted recruitment strategy will be implemented, including organised informational seminars, systematic distribution of flyers, and prominently displayed posters in residential and common areas. Residents who express interest will be invited to provide written informed consent. A mobile screening team will then conduct on-site assessments directly on participants’ residential floors, with screening supervised by trained researchers and geriatric physicians. Portable equipment will be used to diagnose sarcopenia in accordance with the AWGS 2019 criteria, thereby enhancing accessibility for frail older adults. Final eligibility will be determined on the basis of screening results in conjunction with the study’s predefined inclusion and exclusion criteria.

### Randomization and blinding

Reflecting the real-world setting, a cluster randomization design will be employed, in which entire residential buildings within the nursing home will serve as the unit of randomization. A total of 19 residential buildings will be included, each comprising approximately 4–10 eligible participants. Clusters will be allocated as evenly as possible to the four study groups. The randomization sequence will be generated by an independent statistician using SAS 9.4 (SAS, USA), with allocation concealed from recruitment and assessment personnel until assignment [38]. Baseline imbalance at both the individual and cluster levels will be descriptively assessed and considered in adjusted and sensitivity analyses.

Because of the nature of the exercise intervention, participants, caregivers, and exercise supervisors cannot be blinded to group assignment. However, outcome assessors and data analysts will remain blinded wherever feasible. No routine unblinding procedure is planned because the interventions are non-pharmacological and group allocation will be known to intervention providers after randomization.

### Intervention

All participants will first receive a standardized 30-min health education session covering sarcopenia, healthy lifestyle practices, and nutrition-related prevention strategies. Before intervention delivery, caregivers assigned to the exercise groups will undergo centralized training by exercise specialists to ensure standardized implementation across residential buildings. Usual medical care and routine nursing home services will continue throughout the trial in all groups. Participants will be asked not to initiate any additional structured exercise programme outside the study during the intervention period. Any relevant changes in health status, medication use, or rehabilitation participation will be documented where feasible.

To minimize contamination across groups, exercise sessions will be delivered within the assigned residential buildings and scheduled separately by group. Caregivers involved in intervention delivery will be assigned to specific buildings during scheduled sessions, and group-specific exercise materials will not be shared across groups. Participants will be asked not to attend sessions assigned to other groups. Any additional rehabilitation, structured exercise, or cross-group exposure will be recorded through caregiver logs, participant reports, and process evaluation activities [39].

The intervention is designed as a 48-week pragmatic, group-based exercise programme delivered within participants’ residential buildings. Exercise sessions will be conducted three times per week, with each 45-min session comprising a 10-min warm-up, a 30-min core exercise component, and a 5-min relaxation period. To enhance feasibility in routine nursing home care, the programme will be delivered using pre-recorded specialist-led videos and supervised through a phased model in which specialist involvement is progressively reduced and caregiver-led delivery correspondingly increased over time.

During weeks 1–12, specialists will provide on-site supervision for all sessions alongside caregivers. During weeks 13–24, on-site supervision will be reduced to one randomly scheduled visit per week, with the remaining sessions supported remotely. During weeks 25–48, sessions will be primarily supervised by caregivers using the video-guided materials, while specialists continue to provide remote oversight. Attendance at each scheduled exercise session will be recorded electronically by caregivers and summarized as an adherence and feasibility indicator; detailed attendance definitions and calculation procedures are provided in Additional file 1.

Adherence support will be guided by the Capability, Opportunity, Motivation–Behaviour (COM-B) model [40], and the Theoretical Domains Framework (TDF) [41] targeting exercise adherence in participants. Specifically, delivery within participants’ residential buildings, small-group sessions, caregiver assistance, and specialist supervision will target physical and social opportunity; health education, video-guided instruction, and seated exercise formats will target psychological and physical capability; and attendance monitoring, supportive feedback, low-value attendance-based incentives, and public recognition of highly engaged participants who consent to being acknowledged will target reflective and automatic motivation through reinforcement, self-efficacy, social norms, commitment, and habit formation [42].

**Fig. 2:**
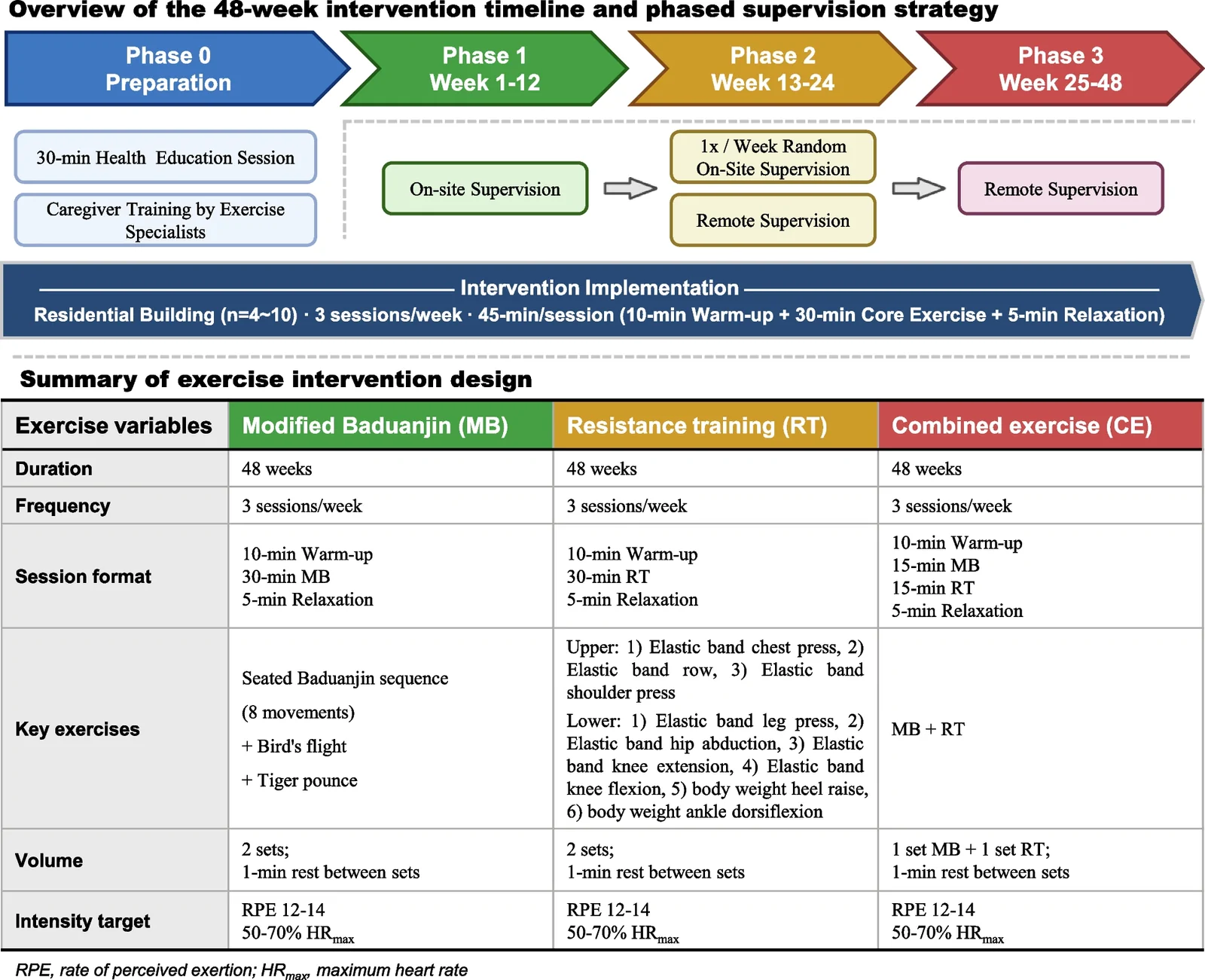
Overview of the 48-week intervention design, implementation timeline, and supervision strategy

Intervention sessions may be modified or temporarily discontinued for individual participants in the presence of acute illness, pain exacerbation, dizziness, chest discomfort, marked fatigue, participant refusal, or any other condition judged by caregivers or supervising staff to make continued exercise unsafe. Participants who discontinue the intervention will be encouraged to remain in outcome assessments whenever possible. Detailed intervention procedures are presented in Fig. 2 and Additional file 1.

#### Modified Baduanjin group

Participants in the modified Baduanjin group will perform a seated, safety-adapted programme developed for oldest-old nursing home residents with limited mobility and elevated fall concerns. The protocol retains the coordinative and mind–body characteristics of traditional Baduanjin while incorporating modifications intended to improve feasibility and lower-limb engagement in frail institutionalised older adults. Detailed exercise protocol and progression rules are provided in Additional file 1.

#### Resistance training group

Participants in the resistance training group will undertake an elastic-band programme targeting major upper- and lower-limb muscle groups relevant to functional mobility and daily activities. The programme is based on established recommendations for resistance exercise in older adults and incorporates progressive adjustment of training load according to participant tolerance and perceived exertion. Detailed exercise protocol and progression rules are provided in Additional file 1.

#### Combined exercise group

Participants in the combined exercise group will receive both modified Baduanjin and resistance training within the same session, with the aim of simultaneously targeting muscular strength, flexibility, and neuromuscular coordination. This combined format is intended to integrate the potential benefits of mind–body exercise and progressive resistance exercise within a feasible nursing home delivery model. To ensure time-matched comparability across groups, the combined exercise group will follow the same session duration and weekly frequency as the single-modality groups. The 30-min core exercise component will comprise approximately 15 min of modified Baduanjin and 15 min of resistance training.

#### Control group

Participants in the control group will attend the same standardized health education session at study initiation but will not receive any study-provided structured exercise during the 48-week intervention period. They will be asked to maintain their usual daily routines and will complete all scheduled assessments concurrently with the intervention groups.

### Outcome assessment

Outcome assessments are scheduled to evaluate both short-term intervention effects and the maintenance of longer-term benefits. With the exception of biospecimen collection, the outcomes will be assessed at baseline, weeks 12, 24, and 48, and again at 1-year and 2-year post-intervention follow-up. HG will additionally be measured every 4 weeks during the intervention period to capture more continuous changes in muscle strength, whereas biospecimens will be collected at baseline and week 48 only. The full assessment schedule is summarized in Table 1.

**Table 1:** Schedule of study assessments

Measure	Screening	Baseline	Intervention												Follow-up	
		**Week 0**	**Week 4**	**Week 8**	**Week 12**	**Week 16**	**Week 20**	**Week 24**	**Week 28**	**Week 32**	**Week 36**	**Week 40**	**Week 44**	**Week 48**	**Year 1**	**Year 2**
Primary outcomes																
Sarcopenia Z-score	** × **	** × **			** × **			** × **						** × **	** × **	** × **
Components																
ASMI	** × **	** × **			** × **			** × **						** × **	** × **	** × **
HG	** × **	** × **	** × **	** × **	** × **	** × **	** × **	** × **	** × **	** × **	** × **	** × **	** × **	** × **	** × **	** × **
SPPB	** × **	** × **			** × **			** × **						** × **	** × **	** × **
Secondary outcomes																
TUG		** × **			** × **			** × **						** × **	** × **	** × **
PA level		** × **			** × **			** × **						** × **	** × **	** × **
sEMG		** × **			** × **			** × **						** × **	** × **	** × **
Balance function		** × **			** × **			** × **						** × **	** × **	** × **
Sample collection		** × **												** × **		
Questionnaires		** × **			** × **			** × **						** × **	** × **	** × **
Clinical events		** × **			** × **			** × **						** × **	** × **	** × **
Care-dependency		** × **			** × **			** × **						** × **	** × **	** × **

“ × ” indicates that the corresponding outcome measure or assessment will be conducted at that visit. Questionnaire-based assessments include the CMMS, MoCA, GDS-15, GAD-7, PSQI, CFS, SF-36, ADL scale, CCMQ, and ACE-based indicators. Repeated questionnaire assessments refer to time-varying domains; non-time-varying or historical variables, including demographic characteristics, educational attainment, family background, and adverse childhood experience indicators, will be collected at baseline only. Clinical events and care-dependency records include mortality, hospitalization or emergency transfer, falls, injurious falls, and care-dependency level, which will be continuously recorded during intervention and follow-up, and will be summarized at baseline, Week 12, Week 24, Week 48, and 1 and 2 years post-intervention

*ASMI* Appendicular Skeletal Muscle Mass Index, *HG* Handgrip Strength, *SPPB* Short Physical Performance Battery, *TUG* Timed Up and Go Test, *PA* Physical Activity, *sEMG* Surface Electromyography, *CMMS *Chinese Mini-Mental Status, *MoCA* Montreal Cognitive Assessment, *GDS-15* 15-item Geriatric Depression Scale, *GAD-7* Generalized Anxiety Disorder-7, *PSQI* Pittsburgh Sleep Quality Index, *CFS* Clinical Frailty Scale, *SF-36* 36-item Short Form Health Survey, *ADL* Activities of Daily Living, *CCMQ* Constitution in Chinese Medicine Questionnaire, *ACE* Adverse Childhood Experiences

All assessments will be conducted by trained research staff using standardized procedures, and outcome assessors will remain blinded to group allocation. To minimize participant burden, assessments will be arranged on residents’ residential floors whenever feasible. Portable and low-risk assessments will be conducted in residents’ rooms, whereas physical performance and device-based assessments will be completed in nearby familiar spaces under safety supervision. Non-time-varying or historical variables will be collected at baseline only. Rest breaks will be provided, and assessments may be split across short sessions, deferred, shortened, or omitted if participants experience fatigue, discomfort, or safety concerns. When the full assessment battery cannot be completed, primary outcome components and safety-related assessments will be prioritized, followed by key functional outcomes; exploratory mechanistic assessments, including sEMG, balance-board testing, and biospecimen collection, will be conducted only when safe and tolerated. Reasons for incomplete assessments will be documented.

#### Baseline characteristics

Baseline characteristics will be collected using a structured electronic questionnaire. Data will be obtained through individualized, face-to-face interviews conducted by trained researchers to ensure accuracy and completeness. The questionnaire will capture detailed information on participants’ demographic characteristics (sex, age, ethnicity, socioeconomic status, educational attainment, and family background), medical history and medication use, social support, cognitive function, psychological well-being, nutritional status, lifestyle and PA habits, fall risk, and pain status.

#### Primary outcome

The primary outcome is a composite sarcopenia Z-score adapted from previous sarcopenia and osteosarcopenia exercise trials that used standardized deficits in muscle mass, muscle strength, and physical performance to provide a continuous measure of sarcopenia severity [31, 43, 44]. In the present study, the score is aligned with the AWGS 2019 diagnostic domains and includes ASMI, HG, and SPPB [34]. These components were selected because they represent the three core domains of sarcopenia: muscle mass, muscle strength, and physical performance. SPPB was chosen as the physical-performance component because it integrates balance, gait speed, and chair-stand performance, which are particularly relevant to frail oldest-old nursing home residents.

$$\begin{aligned} {\mathrm{Z}-\mathrm{s}\mathrm{c}\mathrm{o}\mathrm{r}\mathrm{e}}_{ \mathrm{M}} &=\frac{28.0-\mathrm{i}\mathrm{n}\mathrm{d}\mathrm{i}\mathrm{v}\mathrm{i}\mathrm{d}\mathrm{u}\mathrm{a}\mathrm{l} HG}{\mathrm{S}\mathrm{D} \mathrm{o}\mathrm{f} \mathrm{H}\mathrm{G}}\\ & \quad +\frac{9-\mathrm{i}\mathrm{n}\mathrm{d}\mathrm{i}\mathrm{v}\mathrm{i}\mathrm{d}\mathrm{u}\mathrm{a}\mathrm{l} SPPB}{\mathrm{S}\mathrm{D} \mathrm{o}\mathrm{f} \mathrm{S}\mathrm{P}\mathrm{P}\mathrm{B}}\\ & \quad+\frac{7.0-\mathrm{i}\mathrm{n}\mathrm{d}\mathrm{i}\mathrm{v}\mathrm{i}\mathrm{d}\mathrm{u}\mathrm{a}\mathrm{l} \mathrm{A}\mathrm{S}\mathrm{M}\mathrm{I}}{\mathrm{S}\mathrm{D} \mathrm{o}\mathrm{f} \mathrm{A}\mathrm{S}\mathrm{M}\mathrm{I}} \end{aligned}$$

$$\begin{aligned} {\mathrm{Z}-\mathrm{s}\mathrm{c}\mathrm{o}\mathrm{r}\mathrm{e}}_{ \mathrm{F}}&=\frac{18.0-\mathrm{i}\mathrm{n}\mathrm{d}\mathrm{i}\mathrm{v}\mathrm{i}\mathrm{d}\mathrm{u}\mathrm{a}\mathrm{l} \mathrm{H}\mathrm{G}}{\mathrm{S}\mathrm{D} \mathrm{o}\mathrm{f} HG}\\ & \quad+\frac{9-\mathrm{i}\mathrm{n}\mathrm{d}\mathrm{i}\mathrm{v}\mathrm{i}\mathrm{d}\mathrm{u}\mathrm{a}\mathrm{l} SPPB}{\mathrm{S}\mathrm{D} \mathrm{o}\mathrm{f} \mathrm{S}\mathrm{P}\mathrm{P}\mathrm{B}}\\ & \quad+\frac{5.7-\mathrm{i}\mathrm{n}\mathrm{d}\mathrm{i}\mathrm{v}\mathrm{i}\mathrm{d}\mathrm{u}\mathrm{a}\mathrm{l} \mathrm{A}\mathrm{S}\mathrm{M}\mathrm{I}}{\mathrm{S}\mathrm{D} \mathrm{o}\mathrm{f} \mathrm{A}\mathrm{S}\mathrm{M}\mathrm{I}} \end{aligned}$$

The composite sarcopenia Z-score will be calculated separately by sex based on AWGS 2019. The three standardized deficit components will be summed, with higher scores indicating greater sarcopenia severity. Sex-specific baseline standard deviations will be used for standardization.

1. Muscle mass (ASMI):
  ASMI will be measured using BIA with an InBody 770 device (InBody, Seoul, Korea). Participants will be instructed to remove shoes and socks, stand upright on the device, and maintain an upright posture with arms slightly abducted. Measurements will be performed under standardized conditions, including fasting for at least 4 h and emptying the bladder prior to assessment.
2. Muscle strength (HG):
  HG will be measured using a calibrated Jamar hydraulic hand dynamometer (Patterson Medical, USA). Participants will be seated with the elbow flexed at 90°, forearm in neutral position, and wrist slightly extended. Each hand will be tested three times with a 1-min rest between trials, and the maximum value across all six trials will be recorded in kilograms.
3. Physical performance (SPPB):
  Physical performance will be evaluated using the Short Physical Performance Battery (SPPB), which includes three components: GS test, 5STS test, and balance tests (side-by-side, semi-tandem, tandem). Each component will be scored from 0 to 4 according to standard criteria, with a total SPPB score ranging from 0 (worst) to 12 (best). Assessments will be performed by trained staff following standardized protocols, and participants will be allowed a single practice trial before formal measurements.

#### Secondary outcomes

1. Timed Up and Go Test.
  The Timed Up and Go (TUG) test will be used to assess functional mobility and fall risk [45]. Participants will be instructed to rise from a standard armchair (seat height 43–45 cm), walk at their usual pace for 3 m, turn around, return to the chair, and sit down.
2. Balance Performance.
  Standing balance will be assessed using a modified Clinical Test of Sensory Integration for Balance on a Wii Fit Balance Board (Nintendo Co., Ltd., Kyoto, Japan), with center-of-pressure displacement derived from standardized stance conditions: 1) side-by-side, eyes open, 2) side-by-side, eyes closed and 3) semi-tandem, eyes open [46].
3. Physical Activity and Sedentary Behavior patterns.
  PA and sedentary behaviour will be assessed using both objective monitoring and self-report. Objective data will be collected using a thigh-worn Fibion accelerometer (Fibion Inc., Jyväskylä, Finland) to characterize daily sedentary time and activity intensity [47, 48]. Participants will be asked to wear the device continuously 24 h/day over 7 consecutive days at each assessment time point [49, 50]. A valid assessment will require at least three valid days with a minimum predefined wear duration per day. Self-reported activity and sedentary behaviour over the preceding week will be assessed using the short form of the International Physical Activity Questionnaire (IPAQ-SF) [51].
4. Neuromuscular Activation.
  Neuromuscular activation will be assessed using a wearable textile surface electromyography (sEMG) system (MSleeve, Myontec Ltd., Kuopio, Finland) [52–54]. Recordings from the tibialis anterior, gastrocnemius, and soleus muscles will be obtained during the GS test, 5STS test, TUG test, and standing balance assessment to examine exercise-related changes in lower-limb motor control. Predefined exploratory sEMG variables will include task-specific muscle activation amplitude, activation duration, and, where feasible, muscle co-activation patterns during functional tasks. Raw signals will be processed using standardized filtering, artefact removal, and normalization procedures specified before analysis.
5. Biological Specimen Collection.
  At baseline and week 48, participants will be instructed to provide approximately 5 mL of saliva in a sterile collection tube, excluding froth. Prior to sample collection, participants will be asked to refrain from eating or drinking for 1–2 h to minimize contamination and ensure sample quality, following established saliva collection protocols [55]. All biospecimens will be stored at 4 °C in insulated containers for no more than two hours before transportation to the biochemistry laboratory at Zhejiang University for processing. Saliva samples will be centrifuged at 3,000 rpm for 10 min to separate cellular debris from the supernatant, following standard procedures for saliva sample preparation and biobanking [56]. The resulting fractions will be aliquoted into EP tubes. All processed samples will be stored at -80°C until omics profiling is conducted.
  Saliva-derived DNA will be extracted for telomere length measurement and DNA methylation analyses. Saliva provides a practical and non-invasive source of genomic DNA for large-scale population studies and has been widely used in aging research [57]. Telomere length is a well-established biomarker of cellular aging [58, 59], whereas DNA methylation profiles capture age-related epigenetic alterations and can be used to derive measures of biological aging, such as epigenetic clocks [60, 61]. Integrating these measurements provides complementary information on biological aging processes and may help elucidate mechanisms underlying age-related health outcomes and functional decline [57, 61].
6. Questionnaire-Based Assessments.
  Questionnaire-based assessments will be used to evaluate cognition, mental health, sleep quality, frailty, health-related quality of life, functional independence, traditional Chinese medicine constitution, perceived physical activity needs, and adverse childhood experiences, using validated instruments including the CMMS, MoCA, GDS-15, GAD-7, PSQI, CFS, SF-36, ADL scale, CCMQ, and ACE-based indicators [62–71].
7. Clinical Events and Care-dependency.
  Impaired mobility and sarcopenia-related functional decline are associated with adverse health outcomes, falls, increased healthcare utilization, and care dependency in nursing home residents [72, 73]. Therefore, clinical event records and care-dependency level will be collected through continued collaboration with the nursing home and verified using routine institutional records where available. Baseline clinical event records will capture pre-trial event history, including prior hospitalization or emergency transfer, falls, and injurious falls. During the 48-week intervention period, subsequent clinical events, including all-cause mortality and recorded cause of death, hospitalization or emergency transfer and corresponding reasons, falls, and injurious falls, will be continuously recorded and summarized at Week 12, Week 24, and Week 48. During the post-intervention follow-up period, the same clinical events will continue to be recorded and summarized at 1 and 2 years post intervention. Care-dependency level will be recorded at baseline, Week 12, Week 24, Week 48, and 1 and 2 years post intervention using routine nursing-care registration records [32].

### Feasibility and process evaluation

To complement the effectiveness evaluation, a mixed-methods feasibility and process evaluation will be conducted in accordance with Medical Research Council (MRC) guidance [39]. This component will examine implementation, mechanisms of impact, and contextual factors relevant to delivery within routine nursing home care.

Quantitative indicators will include recruitment, retention, attendance, fidelity, enjoyment, and adverse events, while qualitative data will be collected through interviews, focus groups, field observations, and researcher notes involving residents, caregivers, and administrators. The process evaluation will also explore participants’ and caregivers’ perceptions of the adherence support strategies, including supervision, feedback, incentives, social support and recognition. These data will be used to identify barriers and facilitators to implementation and to inform future refinement of the intervention and trial procedures.

#### Exercise intensity monitoring

Exercise intensity will be monitored to ensure that training is delivered within the prescribed moderate-intensity range. Perceived exertion will be assessed using the Borg Rating of Perceived Exertion scale, with a target range of 12–14. To complement this, heart rate will be measured once for each participant during the intervention period using a Polar OH1 + device (Polar Electro Oy, Kempele, Finland) as an objective spot-check of exercise intensity, with moderate intensity defined as 50–70% of the age-predicted maximum heart rate (HR _Max_ = 208 − 0.7 × age) [74].

### Statistical analysis

All analyses will follow the intention-to-treat (ITT) principle, including all randomized participants in their assigned groups regardless of adherence or withdrawal [75]. Data will be stored in a password-protected database and managed by designated research staff. Analyses will be performed by an independent statistician using R software (R Foundation for Statistical Computing, Vienna, Austria).

Baseline characteristics will be summarized using descriptive statistics. Continuous variables will be presented as mean ± SD or median [IQR], as appropriate, and categorical variables as frequencies and percentages. Baseline balance across groups and clusters will be descriptively assessed.

Primary and secondary continuous outcomes will be analyzed using linear mixed-effects models, with fixed effects for group, time, and group-by-time interaction, and adjustment for the baseline value of the corresponding outcome and relevant covariates where appropriate. Random intercepts for residential building clusters and participants will be included to account for cluster randomization and repeated measurements. The main intervention effect will be estimated from the group-by-time interaction, with Week 48 as the primary endpoint. Bonferroni-adjusted post-hoc comparisons will be conducted where appropriate. Binary or categorical outcomes, including adverse events, will be analyzed using generalized linear mixed models. Long-term clinical events and care-dependency records will be summarized descriptively by group, with exploratory cluster-adjusted analyses conducted if event numbers permit.

Model assumptions will be assessed using residual diagnostics, and transformations or robust methods will be considered if needed. Missing outcome data will be handled using multiple imputation under the missing-at-random assumption. Sensitivity analyses will compare the intention-to-treat results with per-protocol and complete-case analyses. Effect sizes will be reported as standardized mean differences for continuous outcomes and odds ratios for binary outcomes. Statistical significance will be set at a two-sided α = 0.05.

## Discussion

Sarcopenia presents an increasingly critical challenge to healthy ageing, with notably higher prevalence and more severe functional impairments among nursing home residents compared to community-dwelling older adults [76–78]. This vulnerable population experiences significant limitations in physical function, which directly compromise quality of life [5]. While exercise interventions are widely recognised as effective in sarcopenia management, the majority of existing evidence comes from highly controlled trials conducted in community settings [10, 11, 79–82]. This gap in evidence limits our understanding of how these interventions perform in real-world residential care environments.

To address this gap, the SCOPE study focuses on three exercise modalities: modified Baduanjin, resistance training, and their combination. These interventions were selected based on strong evidence from large-scale meta-analyses, which confirm their efficacy in improving sarcopenia-related outcomes [7, 22]. To ensure time-matched comparisons, all intervention groups will receive the same total session duration and weekly frequency. A cluster-randomised design at the residential building level will be employed to align with routine care workflows and minimize intervention contamination, as individual randomization within the same building could lead to cross-contamination between participants and complicate intervention management in nursing home settings. Although residential-building cluster randomization can reduce contamination between trial arms [83], residual contamination cannot be completely ruled out because all clusters are located within one nursing home, where interactions among residents and caregivers occur as part of routine care [84]. Separate building-based delivery, group-specific scheduling and materials, caregiver assignment, and documentation of additional exercise or cross-group exposure are intended to reduce and monitor this risk. Potential contamination will be considered when interpreting between-group effects and explored in sensitivity analyses where data permit [39]. By implementing a caregiver-led, video-assisted delivery model, the SCOPE study is designed to maximise its relevance to routine practice. This approach integrates existing facilities, a flexible supervision schedule, and an adherence incentive system, which will allow assessment of both intervention effectiveness and feasibility, scalability, and sustainability within nursing home settings.

A comprehensive assessment framework will be applied to capture both clinical effectiveness and underlying mechanisms of action. In contrast to prior studies relying on single-domain endpoints [85, 86], the primary outcome is a composite Z-score integrating muscle mass, strength, and physical performance, thereby providing a continuous measure of sarcopenia severity. To account for potential compensatory reductions in daily activity [87], accelerometer-based monitoring will be implemented at multiple time points to quantify overall activity changes and examine their moderating effects [87, 88]. The assessment strategy is further extended to include surface electromyography and center of pressure measures of standing balance in order to elucidate neuromuscular adaptations associated with different exercise modalities [89]. In particular, saliva-derived DNA will be utilized for telomere length determination and methylation analyses, providing accessible biomarkers for aging-related molecular adaptations to exercise [90–92].

Several limitations must be acknowledged. While the SCOPE study will be conducted in the largest nursing home facility in the region, the single-centre design may affect the generalisability of the results. Because reliable ICC and effect-size estimates for the proposed composite sarcopenia Z-score in oldest-old nursing home residents are limited, the sample-size calculation should be interpreted as a pragmatic planning estimate. The study may be underpowered to detect small between-group effects, and the observed ICCs and cluster sizes will be reported to inform future definitive trials. The 48-week intervention period, while necessary for assessing long-term effects and accounting for seasonal variation [93], could increase the risk of attrition due to health deterioration, institutional transfers, or temporary facility closures. To mitigate these challenges, the protocol incorporates adaptive strategies such as intervention period extension, home-based exercise guidance, and optional live-streamed sessions to support continued participation during absences. Additionally, to better simulate real-world staff turnover and resident mobility, the recruitment in predefined waves may be implemented as needed. This flexible approach ensures that the trial can adapt to the dynamic nature of nursing home environments.

Through its cluster-randomised design, the SCOPE study is expected to establish a practical framework for implementing evidence-based exercise programmes in institutional care settings. The findings are expected to inform the development of standardised exercise guidelines for sarcopenia management in long-term care facilities and guide evidence-based decisions regarding staffing and resource allocation. More broadly, the SCOPE study is expected to exemplify the integration of research and practice, providing a scalable model that bridges the gap between controlled efficacy trials and routine care delivery, offering valuable insights for future research aimed at integrating complex interventions into real-world healthcare systems.

## Supplementary Information

Additional file 1. Detailed intervention protocol. This file provides details on caregiver training, phased supervision, warm-up exercises, modified Baduanjin, resistance training, adherence monitoring, exercise intensity monitoring, safety procedures, and intervention fidelity.

Additional file 2. SPIRIT 2025 checklist. This file provides the completed SPIRIT 2025 checklist for the trial protocol.

## Data Availability

No datasets were generated or analysed during the current study. Further information about the SCOPE Study including study progress, related publications, and future updates, will be made available in the SCOPE Study section of the corresponding author's institutional webpage: https://person.zju.edu.cn/en/gaoying.

## Abbreviations

ACE: Adverse Childhood Experiences
ADL: Activities of Daily Living
ASMI: Appendicular Skeletal Muscle Mass Index
AWGS: Asian Working Group for Sarcopenia
BIA: Bioelectrical Impedance Analysis
CCMQ: Constitution in Chinese Medicine Questionnaire
CEG: Combined Exercise Group
CFS: Clinical Frailty Scale
COM-B: Capability, Opportunity, Motivation and Behaviour
CON: Control Group
CV: Coefficient of Variation
GAD-7: Generalized Anxiety Disorder-7
GDS-15: 15-Item Geriatric Depression Scale
GS: Gait Speed
HG: Handgrip Strength
HR _Max_: Maximal Heart Rate
IPAQ-SF: International Physical Activity Questionnaire-Short Form
ICC: Intracluster Correlation Coefficient
ITT: Intention-to-Treat
MBG: Modified Baduanjin Group
CMMS: Chinese Mini-Mental Status
MoCA: Montreal Cognitive Assessment
MRC: Medical Research Council
PA: Physical Activity
PSQI: Pittsburgh Sleep Quality Index
RCT: Randomized Controlled Trial
RTG: Resistance Training Group
sEMG: Surface Electromyography
SF-36: 36-Item Short Form Health Survey
SPPB: Short Physical Performance Battery
TDF: Theoretical Domains Framework
TUG: Timed Up and Go Test
5STS: 5-Time Sit-to-Stand Test
