# Supplementary material for: Implementing an exercise intervention for sarcopenia in oldest-old nursing home residents: protocol for a pragmatic cluster-randomized controlled trial (SCOPE Study)
Source: BMC Geriatr. 2026 Aug 31;26:1154. doi: 10.1186/s12877-026-08002-8 (PMC13560151; doi:10.1186/s12877-026-08002-8)
Supplement: Supplementary file 1 [file 12877_2026_8002_MOESM1_ESM.docx]

**Additional file 1. Detailed intervention protocol**

**1.1 Caregiver training and phased supervision**

Caregivers assigned to the exercise groups will receive centralized standardized training from exercise specialists 1-2 weeks before intervention initiation. Training will include at least three 1-hour sessions and will cover the exercise content, use of video-guided materials, supervision requirements, safety precautions, attendance recording, and participant support during sessions. At the end of training, caregivers will undergo competency evaluation, and only those meeting predefined standards will be involved in intervention delivery.

To enhance feasibility while maintaining intervention fidelity, a phased supervision model will be adopted across the 48-week programme. Specialist involvement will be progressively reduced, while caregiver-led delivery will be correspondingly increased over time (Table S1).

**Table S1. Phased supervision model across the 48-week intervention**

| **Phase** | **Weeks** | **Specialist role** | **Caregiver role** | **Mode of support** |
| --- | --- | --- | --- | --- |
| Phase 1 | Weeks 1-12 | On-site supervision for all sessions | Assist with delivery and participant support | Full in-person specialist supervision |
| Phase 2 | Weeks 13-24 | One randomly scheduled on-site visit per week; remote support for remaining sessions | Lead routine session delivery with support | Hybrid on-site and remote supervision |
| Phase 3 | Weeks 25-48 | Remote oversight | Primary responsibility for session delivery | Caregiver-led implementation with remote specialist support |

**1.2 Warm-up exercise design**

Each intervention session will begin with a standardized 10-minute warm-up designed to enhance joint mobility, stimulate circulation, and prepare participants for the main exercise component. The warm-up routine will comprise walk in place, neck movements, chest expansion, upper-limb stretching, trunk rotation, lateral trunk stretching, wrist movements, heel raises and toe pointing, and ankle circles. All warm-up movements will be performed at low intensity and can be adapted according to participants’ functional status and tolerance (Figure S1).

**Figure S1. Illustrations of Warm-up Exercises.** (a) Walk in Place; (b) Neck Movements; (c) Chest Expansion; (d) Upper-limb Stretching; (e) Trunk Rotation; (f) Lateral Trunk Stretching; (g) Wrist Movements; (h) Heel Raises and Toe Pointing; (i) Ankle Circles

**1.3 Modified Baduanjin design**

In response to the difficulties that oldest-old individuals with sarcopenia may encounter when performing traditional standing Baduanjin exercises [1], as well as the limited lower-limb training stimulus provided by conventional seated forms [2], a progressive modified seated Baduanjin protocol was developed. The design of this protocol was informed by extensive consultations with experts in traditional Chinese martial arts (DW, YXG, and XA) from the Department of Sports Science at Zhejiang University and refined through iterative practice sessions. Drawing on principles from traditional Chinese exercise Wu Qin Xi (the exercise imitating the movements of the tiger, deer, bear, monkey and bird), the modified routine was specifically adapted to accommodate the functional characteristics and safety needs of older adults.

The protocol comprised a structured sequence of seated Baduanjin movements: (1) Holding Up the Sky with Both Hands to Regulate the Sanjiao (Triple Burner), (2) Drawing the Bow to Shoot the Hawk, (3) Lifting the Arms to Regulate the Spleen and Stomach, (4) Looking Backwards to Relieve the Five Fatigues and Seven Injuries, (5) Punching with a Fierce Gaze, (6) Holding the Feet with Two hands to Strengthen the Kidney and Lumbar Area, (7) Clearing the Heart Fire by Shaking the Head and Wagging the Tail, (8) Standing on Tiptoes Eliminates Illnesses, performed as a seated heel-raise and toe-pointing adaptation. Additionally, two traditional seated movements were incorporated: (1) Bird’s Flight, (2) Tiger Pounce. Each exercise will be performed in two sets, with a one-minute rest interval between sets. (Figure S2).

**Figure S2.** **Illustrations of Modified Baduanjin.** (a) Starting Position (Qi Shi); (b) Holding Up the Sky with Both Hands to Regulate the Sanjiao (Triple Burner); (c) Drawing the Bow to Shoot the Hawk (Left and Right); (d) Regulating the Spleen and Stomach by Single Lift; (e) Looking Backwards to Relieve the Five Fatigues and Seven Injuries; (f) Shaking the Head and Wagging the Tail to Clear Heart Fire; (g) Holding the Feet with Two Hands to Strengthen the Kidney and Lumbar Area; (h) Clenching Fists and Scowling to Increase Strength; (i) Standing on Tiptoes to Eliminate Illness; (j) Bird Flying; (k) Tiger Pounce; (l) Ending Position (Shou Shi)

**1.4 Resistance training design**

The resistance training protocol was developed in accordance with the American College of Sports Medicine (ACSM) guidelines for resistance exercise in older adults [3] and was informed by previous studies employing elastic band training in older adults [4-6]. This protocol was designed to enhance strength in the major muscle groups of the trunk, upper limbs, and lower limbs, which are essential for maintaining functional mobility and PA [7].

Following a standardized warm-up, participants will perform a series of upper-body exercises, including (1) seated chest press, (2) seated row, and (3) seated shoulder press, followed by lower-body exercises consisting of (1) leg press movements, (2) hip abduction, (3) knee extension, (4) knee flexion, (5) bodyweight heel raises, and (6) ankle dorsiflexion. Each exercise will be performed for 10 to 20 repetitions across two sets, with a 1-minute rest interval between sets.

Resistance will be provided using the Theratools resistance band system (A&B Physiotherapy, Zhengzhou, China), with different band colours corresponding to progressively increasing resistance levels (ranging from 10, 15, 20, 25 and 30 pounds). Training will commence with the lightest band for 10 repetitions [8]. When participants are able to complete 20 repetitions at a perceived exertion level of 12 on the Borg Rating of Perceived Exertion (RPE) 6-20 scale, resistance will be increased by progressing to the next band level. If 10 repetitions cannot be maintained at the higher resistance, the previous band will be reinstated and repetition number will be gradually increased until the training load can be sustained [7].

**Figure S3. Illustrations of Resistance Training.** (a) Elastic Band Chest Press; (b) Elastic Band Row; (c) Elastic Band Shoulder Press; (d) Elastic Band Leg Press; (e) Elastic Band Hip Abduction; (f) Elastic Band Leg Extension; (g) Elastic Band Leg Curl; (h) Body Weight Heel Raise; (i) Body Weight Ankle Dorsiflexion

**1.5 Combined exercise design**

Participants in the combined exercise group will receive the same total session duration and weekly frequency as the single-modality groups. Each 45-min session will include a 10-min warm-up, a 30-min core exercise component, and a 5-min relaxation period. The 30-min core component will be divided approximately equally between modified Baduanjin and resistance-training elements, thereby providing an integrated but time-matched multimodal programme.

**1.6 Adherence, exercise intensity, and safety monitoring**

**(1) Adherence monitoring**

Attendance will be recorded electronically for each exercise session. Sessions lasting less than 30 minutes will be classified as incomplete for adherence calculation, while partial attendance will also be recorded for process evaluation. Overall attendance will be calculated as the number of completed sessions divided by the number of planned sessions multiplied by 100%. To support adherence, participants with monthly attendance exceeding 85% will be eligible to redeem daily necessities as incentives.

**Table S2. Adherence monitoring procedures**

| **Item** | **Procedure** |
| --- | --- |
| Attendance recording | Recorded electronically for each scheduled session |
| Minimum session completion | Classified as incomplete and not counted as a completed session |
| Attendance calculation | Completed sessions / planned sessions × 100% |
| Incentive criterion | Monthly attendance >85% |

**(2) Exercise intensity monitoring**

Exercise intensity will be monitored using both subjective and objective methods. At the end of each exercise session (warm-up session and two core exercise sessions), participants will report perceived exertion using the Borg Rating of Perceived Exertion scale, with a target range of 12-14. In addition, heart rate will be measured as an objective spot-check of exercise intensity during the intervention period in each participant using Polar OH1+ devices (Polar Electro Oy, Kempele, Finland), with the target intensity defined as 50-70% of age-predicted maximum heart rate. Age-predicted maximum heart rate will be estimated as 208 − 0.7 × age[9].

**(3) Safety monitoring**

Participants will be observed by caregivers and/or exercise specialists during sessions according to the supervision phase. If dizziness, chest discomfort, pain, excessive fatigue, or other adverse symptoms occur during training, the session will be immediately interrupted and the event will be documented by the research team, including symptom type, timing, severity, action taken, and outcome. Participants requiring further evaluation will be referred to clinical staff according to institutional procedures.

**(4) Intervention fidelity**

Intervention fidelity will be assessed using a standardized checklist during on-site supervision and selected remote observations. Fidelity indicators will include adherence to session structure, correct exercise delivery, appropriate progression or modification, safety monitoring, and completion of attendance records. Deviations will be documented and addressed through feedback or retraining.

**References**

1.	Xu S, Gong Z, Wang F, Cao M, Liu J, Chen C, Zhang N, Kang J, Xu C, Peng N: **Intervention of muscle-building and antifrailty exercise combined with Baduanjin for frailty of different functional levels: study protocol for a randomised controlled trial**. *BMJ Open* 2023, **13**(12):e074827.

2.	Chen CF, Chiu SC, Chiang CK, Lu YW: **Effects of seated Baduanjin training modalities on body composition, muscle strength, and gait parameters in older adults: A cluster-randomised controlled trial**. *Complement Ther Med* 2025, **95**:103300.

3.	Nelson ME, Rejeski WJ, Blair SN, Duncan PW, Judge JO, King AC, Macera CA, Castaneda-Sceppa C: **Physical activity and public health in older adults: recommendation from the American College of Sports Medicine and the American Heart Association**. *Med Sci Sports Exerc* 2007, **39**(8):1435-1445.

4.	Yoon DH, Lee JY, Song W: **Effects of Resistance Exercise Training on Cognitive Function and Physical Performance in Cognitive Frailty: A Randomized Controlled Trial**. *J Nutr Health Aging* 2018, **22**(8):944-951.

5.	Valdés-Badilla P, Guzmán-Muñoz E, Hernandez-Martinez J, Núñez-Espinosa C, Delgado-Floody P, Herrera-Valenzuela T, Branco BHM, Zapata-Bastias J, Nobari H: **Effectiveness of elastic band training and group-based dance on physical-functional performance in older women with sarcopenia: a pilot study**. *BMC Public Health* 2023, **23**(1):2113.

6.	Shoepe TC, Ramirez DA, Rovetti RJ, Kohler DR, Almstedt HC: **The Effects of 24 weeks of Resistance Training with Simultaneous Elastic and Free Weight Loading on Muscular Performance of Novice Lifters**. *J Hum Kinet* 2011, **29**:93-106.

7.	Liao CD, Tsauo JY, Huang SW, Ku JW, Hsiao DJ, Liou TH: **Effects of elastic band exercise on lean mass and physical capacity in older women with sarcopenic obesity: A randomized controlled trial**. *Sci Rep* 2018, **8**(1):2317.

8.	Wang SS, Cui M, Li JY, Zhang HM, Liu XY, Han J: **Ameliorated lipid distribution in prediabetes - Effects of 12 weeks traditional Chinese YiJinJing exercise plus TheraBand: A randomized controlled trial**. *J Exerc Sci Fit* 2024, **22**(4):438-444.

9.	Tanaka H, Monahan KD, Seals DR: **Age-predicted maximal heart rate revisited**. *J Am Coll Cardiol* 2001, **37**(1):153-156.
